# Simultaneous Measurement of IgM and IgG Antibodies to SARS-CoV-2 Spike, RBD, and Nucleocapsid Multiplexed in a Single Assay on the xMAP INTELLIFLEX DR-SE Flow Analyzer

**DOI:** 10.1128/spectrum.02507-21

**Published:** 2022-04-07

**Authors:** Andrew Cameron, Jessica L. Bohrhunter, Claire A. Porterfield, Rupinder Mangat, Michael H. Karasick, Zachary Pearson, Stephen Angeloni, Nicole D. Pecora

**Affiliations:** a Pathology and Laboratory Medicine, University of Rochester Medical Center, Rochester, New York, USA; b Division of Infectious Diseases, Department of Medicine, University of Rochester Medical Center, Rochester, New York, USA; c Department of Pathology, Brigham and Women’s Hospital, Harvard Medical School, Boston, Massachusetts, USA; d Luminex Corporation, Austin, Texas, USA; Kumamoto University

**Keywords:** COVID-19, SARS-CoV-2, antibody, nucleocapsid, spike glycoprotein, ACE2, fluorescent microsphere immunoassay, FMIA, microsphere, serology

## Abstract

The multiplex capabilities of the new xMAP INTELLIFLEX DR-SE flow analyzer were explored by modifying a serological assay previously used to characterize the IgG antibody to infection with severe acute respiratory syndrome coronavirus 2 (SARS-CoV-2). The goal was to examine the instrument’s performance and to simultaneously measure IgM and IgG antibody responses against multiple SARS-CoV-2 antigens in a single assay. Specific antibodies against the SARS-CoV-2 spike (S), receptor binding domain (RBD), and nucleocapsid (N) proteins were investigated in 310 symptomatic case patients using a fluorescent microsphere immunoassay and simultaneous detection of IgM and IgG. Neutralization potential was studied using the addition of soluble angiotensin-converting enzyme 2 (ACE2) to block antibody binding. A profile extending to 180 days from symptom onset (DFSO) was described for antibodies specific to each viral antigen. Generally, IgM levels peaked and declined rapidly ∼3–4 weeks following infection, whereas S- and RBD-specific IgG plateaued at 80 DFSO. ACE2 more effectively prevented IgM and IgG binding in convalescent cases > 30 DFSO, suggesting those antibodies had greater neutralization potential. This work highlighted the multiplex and multi-analyte potential of the xMAP INTELLIFLEX DR-SE, and provided further evidence for antigen-specific IgM and IgG trajectories in acute and convalescent cases.

**IMPORTANCE** The xMAP INTELLIFLEX DR-SE enabled simultaneous and semi-quantitative detection of both IgM and IgG to three different SARS-CoV-2 antigens in a single assay. The assay format is advantageous for rapid and medium-throughput profiling using a small volume of specimen. The xMAP INTELLIFLEX DR-SE technology demonstrated the potential to include numerous SARS-CoV-2 antigens; future work could incorporate multiple spike protein variants in a single assay. This could be an important feature for assessing the serological response to emerging variants of SARS-CoV-2.

## INTRODUCTION

Although molecular testing remains the gold standard for the diagnosis of SARS-CoV-2 (1), serological information can provide diagnostic support, infection management, and risk mitigation at the individual level, and infection surveillance at the population level (1). The intensification of worldwide vaccination efforts in the fight against the COVID-19 pandemic may place future importance on serological testing for the discrimination between vaccinated, infected, or susceptible individuals. Furthermore, in the light of the SARS-CoV-2 variants of concern (VOC) in current global circulation (2, 3), the kinetics, quality, and neutralization potential of the serological response to specific variants may be of continued interest as novel mutations emerge.

The structural SARS-CoV-2 spike glycoprotein (S) and the nucleocapsid protein (N) appear to be prime targets of the antibody response (4). Most antibodies that neutralize SARS-CoV-2 and decrease infectivity bind the receptor binding domain (RBD) of S, a consequence of RBD’s interaction with host angiotensin-converting enzyme 2 (ACE2) during viral entry into the host cell (5). Neutralizing antibodies appear to correlate with anti-S antibody levels and may persist for months (6). Due to its key role in inducing neutralizing antibodies, all current viral vector and mRNA vaccines are based on the expression of S protein (7).

There are numerous marketed serological tests capable of detecting various antibody isotypes (e.g., total antibody, IgM, IgG, IgA) to the major SARS-CoV-2 antigens, including S, RBD, and N (8). These assays are typically run in parallel or reflexed, or are otherwise incapable of simultaneous differentiation between isotypes. Previously, a research-use-only multiplex fluorescent microsphere immunoassay (FMIA) assay was developed for measuring the IgG response to the SARS-CoV-2 S, RBD, and N antigens, and was found to be highly sensitive and specific (9). In this study, we modified the assay to detect both IgM and IgG to be performed on a new “dual reporter” instrument (INTELLIFLEX DR-SE) that can measure two fluorescent signals or analytes at the same time. We evaluated the instrument’s performance and “dual reporter” capabilities to simultaneously measure IgG and IgM antibody levels, and also neutralizing potential to the SARS-CoV-2 S, RBD, and N viral antigens in one reaction.

We demonstrate here the potential of the INTELLIFLEX DR-SE as a robust platform for measuring both changes in analyte levels as well as the degree of target-specific neutralization by profiling the antibody response to SARS-CoV-2 infection in acute and convalescent cases, spanning up to 180 days from symptom onset (DFSO).

## RESULTS

### Case demographics.

Of the 310 unique patients that comprised the SARS-CoV-2 cases in this study, 174 were women and 136 were men (Data Set S1 in the supplemental material). The mean ages were 54.6 and 56.8 years, respectively. The rate of ICU admission was higher in men (56/136 [41.0%]) than in women (47/174 [27.0%]). The mean age of ICU versus non-ICU patients was 62.1 and 53.2 years for men compared to 68.3 and 49.4 years for women, respectively.

### Characterization and duration of the longitudinal IgM and IgG serological response to SARS-CoV-2.

**FIG 1:**
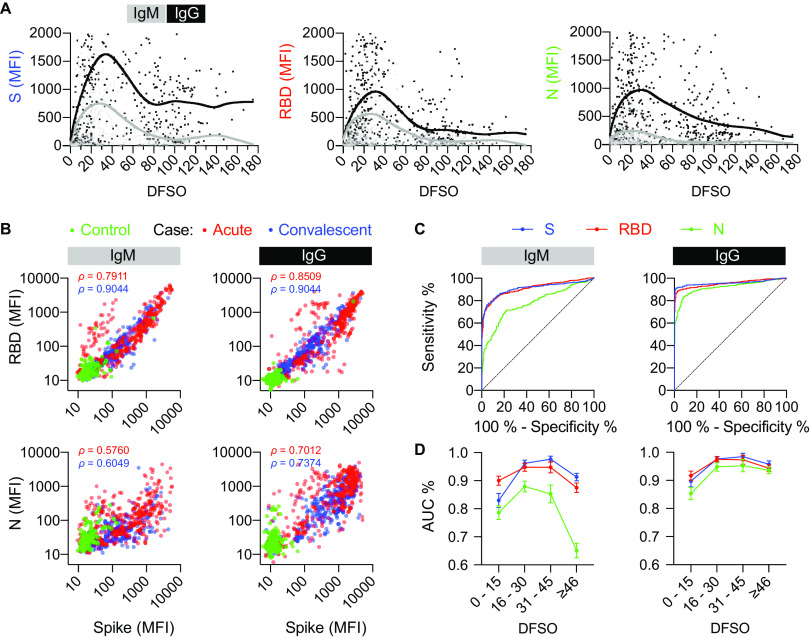
Multi-analyte interrogation of serum from SARS-CoV-2-infected patients. (A) Median fluorescent intensity (MFI) values with lowess curves for IgG (black) and IgM (gray) analytes detected in SARS-CoV-2-positive patient serum. Each circle represents the MFI of a unique sample (*n *= 544, from 310 unique patients). MFI values are shown by days from symptom onset (DFSO) separately for each antigen (spike [S, left panel], spike receptor binding domain [RBD, middle panel], and nucleocapsid [N, right panel]). (B) MFI values for IgM and IgG and correlation coefficients (Spearman *ρ*) for RBD (*y* axis, upper) and N (*y* axis, lower) versus S (*x* axis) antigens. Samples shown are controls (pre-COVID-19 [*n *= 142, all from unique patients] or negative by NAAT or serology [*n* = 122]) and SARS-CoV-2-infected cases. Case sera (*n *= 544) are depicted as acute (collected ≤ 30 DFSO [*n *= 280, from 143 unique patients comprising 66 women and 77 men, with an average age of 64.3 years]) or convalescent (collected > 30 DFSO [*n *= 264, from 196 unique patients comprising of 121 women and 74 men, with an average age of 53.2 years]). (C) Receiver operating curves for each isotype against S, RBD, and N viral antigens. (D) Performance of IgM and IgG for classifying controls and cases stratified by antigen and by DFSO (area under the receiver operating curve [AUC]).

MFI values for IgM and IgG to the S, RBD, and N viral antigens (Data Set S1) were plotted with respect to DFSO (Fig. 1A). The peak IgM response for S, RBD, and N occurred on 28.0, 26.0, and 21.0 DFSO, respectively. The peak IgG response occurred on 33.9, 30.9, and 30.1 DFSO, respectively. Collectively, for IgM, the greatest mean MFI values were found in the 16–30 DFSO group, whereas the greatest mean MFI values for IgG were observed 31–45 DFSO. Thus, IgM specific for each viral antigen declined first, but both IgM and IgG declined ≥46 DFSO (Table 1).

**TABLE 1:** Mean IgM and IgG MFI values for detection of SARS-CoV-2 S, RBD, and N antigens

Sample type		IgM						IgG					
		S		RBD		N		S		RBD		N	
	*n*	MFI	Ratio^*a*^	MFI	Ratio^*a*^	MFI	Ratio^*a*^	MFI	Ratio^*a*^	MFI	Ratio^*a*^	MFI	Ratio^*a*^
Controls													
All controls^*b*^	164	22.5		21.4		38.1		13.1		11.5		32.2	
Cutoffs^*c*^		>135.6		>149.4		>283.1		>95.1		>87.5		>574.1	
Non-ICU cases													
DFSO 0–15	56	423.1		303.0		91.4		579.6		264.7		547.9	
DFSO 16–30	35	923.1		750.9		202.7		1223.1		552.8		1120.2	
DFSO 31–45	19	773.6		484.0		132.6		1507.0		697.4		883.6	
DFSO ≥ 46	184	156.2		112.9		47.6		810.0		301.3		455.7	
ICU cases													
DFSO 0–15	90	432.6	(1.0)	445.6	(1.5)	210.6	(2.3)	733.8	(1.3)	674.7	(2.5)	815.2	(1.5)
DFSO 16–30	99	993.9	(1.1)	767.5	(1.0)	417.7	(2.1)	1874.5	(1.5)	1326.0	(2.4)	1289.9	(1.2)
DFSO 31–45	35	889.5	(1.1)	586.8	(1.2)	290.8	(2.2)	2331.2	(1.5)	1406.6	(2.0)	1091.7	(1.2)
DFSO ≥ 46	26	293.9	(1.9)	211.1	(1.9)	70.5	(1.5)	1176.6	(1.5)	527.9	(1.8)	584.0	(1.3)

a Ratio calculated for ICU versus non-ICU admitted patients only.

b Negative by NAAT or serology for SARS-CoV-2.

c MFI cutoff determined for 100% specificity.

### ICU cases show increased antibody MFIs and IgM and IgG skewed toward different viral antigens.

Sera from ICU-admitted patients was found to have higher mean MFI values for all analytes and antigens than non-ICU cases in all binned groups (0–15, 16–30, 31–45, and ≥46 DFSO; Table 1). To assess for prominent differences, the ratio of the mean MFI for ICU versus non-ICU was compared for each analyte and antigen (Table 1). For ICU versus non-ICU cases, the ratio of IgM directed against N was highest (>2.0, [ICU/non-ICU]) in all bins from 0–45 DFSO. In contrast, the ratio of RBD-specific IgG was greatest (>2.0) in all bins from 0–45 DFSO.

### Antibody levels for S and RBD correlate more closely in convalescence.

To assess how the different antibody responses paralleled each other, pairwise correlations between the MFI values for RBD and N versus S were performed. For all viral antigens, both IgM and IgG MFI values correlated more closely in convalescent cases (Fig. 1B). In all cases, MFI values for RBD and S correlated more closely than MFI values for N and S. The highest correlation was seen between IgG specific for RBD and S in convalescent cases (ρ = 0.90, CI: 0.88 to 0.92). The lowest correlation was between IgM specific for N and S in acute cases (ρ = 0.58, CI: 0.50 to 0.65).

### Comparative performance of IgM and IgG dual detection with multiplexed SARS-CoV-2 antigens.

Performance for each combination of antigen (S, RBD, N) and analyte (IgM, IgG) was assessed by receiver operator characteristic (ROC) curves for 264 controls and 544 cases. For IgM, the AUC for S was 0.92 (CI: 0.90 to 0.94), for RBD it was 0.91 (CI: 0.90 to 0.93), and for N it was 0.78 (CI: 0.74 to 0.81) (Fig. 1C, left). For IgG, the AUC for S was 0.95 (CI: 0.94 to 0.97), for RBD it was 0.95 (CI: 0.94 to 0.97), and for N it was 0.93 (CI: 0.91 to 0.94) (Fig. 1C, right). There was a significant difference between the AUCs for IgM and IgG: IgG outperformed IgM for all antigens detected (*P* < 0.0001, Tukey’s multiple-comparison test). IgM performance was significantly different between the S-based antigens (S, RBD) and N (*P* <0.0001).

Separately, assay performance along the infection time course was calculated for cases binned between 0–15 (*n *= 146), 16–30 (*n *= 134), 31–45 (*n *= 54), and ≥46 DFSO (*n *= 210) (Fig. 1D). For both IgM and IgG, S had the greatest AUC when detected at 31–45 DFSO (IgM: 0.98 [CI: 0.95 to 1.00]; IgG: 0.98 [CI: 0.96 to 1.00]). Of note, IgM detection of N gave the poorest performance in each binned time point, and the AUC declined at ≥46 DFSO to 0.67 (CI: 0.62 to 0.72).

Using the ROC curve, MFI cut-offs and sensitivity were calculated for an assay with 100% specificity for each antigen/analyte combination (i.e., cut-offs eliminating the possibility of false-positives in the control group). For 100% specificity with respect to IgM, the MFI cutoff and sensitivity for S was >135.6 MFI and 54.8%; for RBD it was >149.4 MFI and 44.1%; and for N it was >283.1 MFI and 13.1%. For 100% specificity with respect to IgG, the MFI cutoff and sensitivity for S was >95.1 MFI and 81.6%; for RBD it was >87.5 MFI and 69.7%; and for N it was >574.1 MFI and 41.5%.

### Convalescent cases exhibit increased ACE2-mediated neutralization of IgG binding to S and RBD.

**FIG 2:**
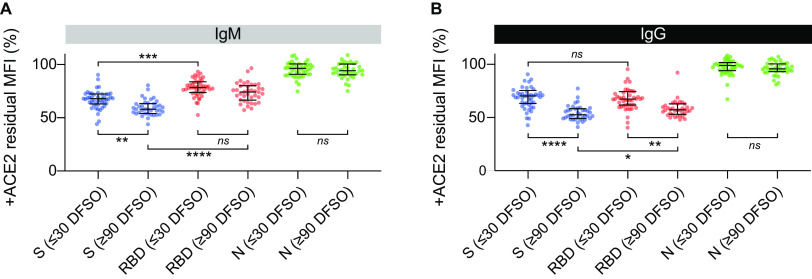
ACE2 neutralization of IgM and IgG binding using acute and convalescent case serum. The neutralizing effect was expressed as the MFI measured with ACE2 as percentage of the MFI measured without ACE2 (residual MFI %). Residual MFI % detected for (A) IgM and (B) IgG in unique patient samples collected ≤30 DFSO (*n *= 48) or ≥90 DFSO (*n *= 38).

The effect of ACE2 addition on the measurement of IgM and IgG bound to each viral antigen was assessed by incubating the antigen-coupled microspheres with soluble ACE2 prior to the addition of case sera. The effect was expressed using the MFI measured for microspheres incubated with ACE2 as a percentage of the MFI measured for microspheres without ACE2 (residual MFI %). For both IgM (Fig. 2A) and IgG (Fig. 2B), the addition of ACE2 resulted in significant decreases in residual MFI % for S and RBD (all *P* < 0.0001, compared to N) for sera from both acute (≤30 DFSO) and convalescent cases (≥90 DFSO). No differences were seen for N-specific IgM and IgG MFIs from either acute or convalescent cases following the addition of ACE2.

For IgM, there was a significant decrease in the residual MFI % for S in convalescent cases compared to acute cases (*P = *0.0089), which was not observed for RBD (*P = *0.1068). The residual MFI % for S-specific IgM was lower than RBD-specific IgM in both acute and convalescent cases (all *P* ≤ 0.001). For IgG, the residual MFI % was significantly lower in convalescent cases, and was lower for both S and RBD (all *P < *0.01). Residual MFI % was not significantly different between S- and RBD-specific IgG for acute cases (*P = *0.1463), but the effect of ACE2 addition was greater on RBD-specific IgG than S-specific IgG in convalescent cases (*P = *0.0289).

## DISCUSSION

In this investigation, we demonstrated the relative ease of modifying a previously described Luminex xMAP-based serological assay (9) for performance on the xMAP INTELLIFLEX DR-SE flow analyzer (10), enabling the measurement of two fluorescent reporters—assigned here to specific detection of IgM and IgG. Thus, multiplexing on the new instrument facilitated the simultaneous and semi-quantitative detection of different antibody classes to 3 different SARS-CoV-2 antigens in a single assay, which was advantageous for rapid and medium-throughput profiling using a small volume of each specimen. Advantages of this approach include the future incorporation of additional antigens (e.g., S variant proteins) on different fluorescent beads, enabling discrimination of specific antibody. A disadvantage of this approach is the manual nature of the assay with respect to reagent preparation, washing, and analysis.

Over the course of the pandemic, though simultaneous yet differentiated detection has not been widely utilized, a growing body of literature indicates that antibodies against SARS-CoV-2 appear 5–7 days after infection, with IgM and IgG raised virtually in parallel (11). The relative antigen kinetics reported here have been observed by others (12, 13), and were also described by us in a previous study utilizing a different instrument (9). Using the dual reporter assay, our data supports this and also the observation that IgM levels peak and decline rapidly (around 3–4 weeks following infection), with IgM peaks tending to occur earlier than IgG (14). With the exception of N-specific IgM, we found no remarkable differences in the time-to-peak between the different SARS-CoV-2 antigens, but observed that IgM markedly declines, especially N-specific IgM.

Although we did not establish this as a clinical assay, the majority of cases tested using only IgM and N would have been considered negative at ≥46 DFSO using MFI cutoffs designed for a 100% specific assay. This was pronounced in outpatient cases of COVID-19, which are predicted to be milder. Other studies have previously demonstrated that severe COVID-19 correlates with higher antibody levels to SARS-CoV-2 antigens (14), which we also observed among patients requiring ICU admission. In this study, IgG levels specific to S and RBD appeared to stabilize at approximately 80 DFSO, and were detected at a similar level at approximately 180 DFSO. However, our conclusions are limited given the bulk of these convalescent samples were obtained between 80 and 100 DFSO with relatively few samples beyond that time.

The polyclonal nature of the early humoral response (15) likely contributes to our observation that IgG (and to a lesser extent, IgM) MFI values for each antigen correlated more closely in convalescent than acute cases. Since RBD is a part of S, we expected RBD-specific antibody responses to follow the same trajectory as S-specific antibody (and less so with N-specific antibody). This tendency was observed, although overall MFI levels were different between each antigen. Differences in MFI levels between antigen is likely due to the fact that S was coupled to the beads at 10 pM, while N and RBD were coupled at 100 pM.

Although IgM secreted following infection is antigen-specific, to a limited degree it may demonstrate cross-reactivity with other antigens. Overall, we found that IgM performance was inferior to IgG when classifying cases and controls, and N-specific IgM performed particularly poorly. It’s not clear as to whether this is because IgM levels decrease relatively rapidly following infections, or if this finding is an artifact of this multiplex assay. Although N antibodies are presumably not protective, and S-targeted immunity is associated with protection (15), N is still immunogenic and N-specific antibodies are produced concurrently with S-specific antibodies. Nevertheless, assaying N-specific IgM did not classify cases and controls as accurately as S- and RBD-specific IgM, or compared to IgG.

Clinical outcome correlations with S-, RBD-, or N-skewed antibody trajectories have been observed, with some studies suggesting compromised development of S-specific antibodies and stronger N-specific responses in mortalities (15), or S- and RBD-focused responses associated with milder infections (16). Our data demonstrated similar skews; comparing MFI ratios between ICU- and nonadmitted cases, N-specific IgM was 2.3-fold higher in early (0–15 DFSO) ICU cases, whereas RBD-specific IgG was 2.5-fold higher in early ICU cases. The significance of this for prognosis is unclear because of the differences in platforms and the semi-quantitative nature of this assay.

Perhaps a more important factor is the development of neutralizing antibodies. To our knowledge, there are few other similar studies presenting data in the form of % inhibition of ACE2 binding. In our previous study we noted variability between individuals over time (9), which has also been shown by others using different assay formats (12, 17, 18). The data in the current study suggested that, in aggregate across multiple individuals, ACE2 more effectively prevented IgM and IgG binding in convalescent cases, which we interpret as indicative of a greater proportion of neutralizing antibodies. This trend toward greater % inhibition by ACE2 (which was specific for S and RBD) may indicate enhanced avidity, which has been demonstrated by others (19).

There are several limitations of this study. The cases here were sampled irregularly from a convenience cohort. Although infrequent, high MFI values measured in several control samples—notably for anti-N antibodies—affected the sensitivity and classification of cases and controls. It may be unlikely that these high MFI values were due to past infections with SARS coronaviruses or seasonal coronavirus; however, these control samples included specimens that had been submitted as part of routine clinical testing for a variety of infectious diseases, including Lyme disease, syphilis, cytomegalovirus, and Epstein-Barr virus, as well as testing for autoimmune markers, such as antinuclear antigen and rheumatoid factor. Therefore, the control samples may represent individuals producing nonspecific antibodies. Pediatric and asymptomatic cases were not in this data set, meaning that these findings are limited to the described patient population.

Another limitation is that case serum was collected from a time (i.e., between April and September 2020) that predated widespread efforts to characterize SARS-CoV-2 VOC. While we cannot preclude that any of the serum collected for this study was from individuals infected with a VOC, epidemiological data from our region does not support widespread circulation of major variants at that time. Thus, it is unknown how this assay would perform in the context of current variants, such as the Delta VOCs, and it would be of future interest to characterize the detection and neutralization potential of antibodies from SARS-CoV-2 VOC cases using a multiplex assay incorporating variant S proteins.

In conclusion, we have evaluated the multiplex capabilities of a novel flow analyzer by reconfiguring a previous FMIA for SARS-CoV-2, and have simultaneously described the IgM and IgG antibody response in our COVID-19 case population.

## MATERIALS AND METHODS

### Clinical laboratory, patient population, and chart review.

This study was conducted using residual patient sera under University of Rochester (UR) Institutional Review Board approval RSRB STUDY00005117. The UR Medicine clinical microbiology laboratory serves hospitals, practices, nursing homes, and urgent cares in Rochester and several counties in Western NY. Patient charts were reviewed twice by a three-person team and days from symptom onset (DFSO) were established for 310 symptomatic patients who had previously tested positive for SARS-CoV-2 by either a nucleic acid amplification test (NAAT) or serology (Abbott Architect SARS-CoV-2 IgG). Of these, 154 had serum collected from routine clinical care as admitted inpatients (including nursing home patients), and 156 were collected from outpatient visits. Sera from inpatients were typically collected earlier (25.1 DFSO ± 28.6, mean ± SD) in the course of infection than from outpatients (73.2 DFSO ± 34.9). Case sera (*n *= 544) were collected between April 2020 and September 2020, stored frozen at −20°C, and thawed at 4°C prior to testing. Controls (*n *= 264) include both pre-COVID-19 sera (*n *= 142, collected between July 2019 and February 2020) and sera from patients with no history (i.e., negative by NAAT or by serology) of COVID-19 (*n *= 122, collected April 2020–June 2020). All patient information was de-identified prior to analysis.

### Multiplex microsphere-based immunoassay.

As previously described (9), antigen-conjugated microspheres/beads were coupled to viral proteins (GenScript Biotech Corporation, Piscataway, NJ) according to the manufacturer’s instructions. The extracellular domain of the S protein (catalog no. Z03481-100), RBD (catalog no. Z03483-100), and the full-length SARS-CoV-2 N protein (catalog no. Z03480-100), were all purchased from GenScript Biotech Corporation (Piscataway, NJ). S protein was coupled at 10 pM, while N and RBD were coupled at 100 pM (i.e., equimolar coupling). For controls, an internal control bead (IC45) and beads separately coupled with human IgG and IgM were also included in each multiplexed (6-plex) assay.

For the assay, 6-plex bead mixes (2,500 beads/ea) were incubated in black polystyrene flat-bottom 96-well assay plates (Corning, Corning, NY, catalog no. 3650) with serum diluted 1:2,000 in PBS-TBN (phosphate-buffered saline [PBS], 0.02%; Tween, 0.1%; bovine serum albumin [BSA], 1%; azide, 0.05%) for 15 min at 37°C. Beads were retained by magnetic separation and washed twice with 150 μL of PBS-TBN by shaking for 2 min at 37°C. For detection, beads were incubated with 100 μL of conjugate for 15 min at 37°C (1.2 μg/mL biotin-goat-α-Human IgG, Fcγ fragment specific [Jackson ImmunoResearch, West Grove, PA, catalog no. 109-065-098], 8.0 μg/mL streptavidin-SuperBright 436 [Invitrogen, catalog no. 62-4317-82], and 1.0 μg/mL of phycoerythrin-conjugated AffiniPure F(ab')_2_ fragment goat α-human IgM Fc_5μ_-fragment-specific [Jackson ImmunoResearch, catalog no. 09-116-129]). As before, beads were retained by magnetic separation and washed twice with 150 μL of PBS-TBN. Finally, beads were resuspended in 100 μL PBS-TBN. The assay was read on the xMAP INTELLIFLEX DR-SE flow analyzer (Luminex) with the following settings: count volume = 50 μL; minimum bead count = 100 beads. Fluorescence was reported as median fluorescent intensity (MFI) values (Data Set S1).

### ACE2 inhibition.

The effect of pre-incubating ACE2 (AdipoGen Corporation, San Diego, CA, catalog no. 0192-30) with the 6-plex bead mix was compared to a control without ACE2 added. The 6-plex bead mix was incubated ± 10.7 μg/mL ACE2 for 2 min at 37°C with shaking (the final ACE2 concentration is 8 μg/mL after the addition of serum). To assess for differences between acute versus convalescent samples, sera was collected for two groups: either from unique cases all within 30 DFSO (*n *= 48), or those ≥ 90 DFSO (*n *= 38). The ACE2 effect was expressed as the % residual MFI detected (i.e., MFI [with ACE2 incubation]/MFI [without ACE2 incubation] × 100).

### Statistical analysis.

Graphical and statistical analysis was performed in Prism 8 (GraphPad Software, San Diego, CA). Unless otherwise indicated, error bars indicate mean ± standard error of the mean (SEM). The area under the receiver operator curve (AUC, expressed as a percentage) was calculated for all controls and cases, or for case serum binned into groups by collection between 0–15, 16–30, 31–45, and ≥46 DFSO.
